# Hyperhomocysteinemia and Disease—Is 10 μmol/L a Suitable New Threshold Limit?

**DOI:** 10.3390/ijms252212295

**Published:** 2024-11-15

**Authors:** Giada Marroncini, Serena Martinelli, Sara Menchetti, Francesco Bombardiere, Francesco Saverio Martelli

**Affiliations:** 1 Biomolecular Diagnostic Laboratories, Via N. Porpora, 50144 Florence, Italy; sara.menchetti@bdmail.it (S.M.); francesco.bombardiere@biomoleculardiagnostic.com (F.B.); francesco.martelli@ednmail.it (F.S.M.); 2 Department of Clinical and Experimental Medicine, University of Florence, 50139 Florence, Italy; serena.martinelli@unifi.it

**Keywords:** hyperhomocysteinemia, reference range, cardiovascular disease, neurological disease, osteoporosis and cancer

## Abstract

Hyperhomocysteinemia (HHcy) is a medical condition characterized by an abnormally high level of homocysteine (Hcy) in the blood. Homocysteine is a toxic sulfur-containing amino acid that is produced during the metabolism of methionine. Under normal circumstances, Hcy is recycled back to methionine via the remethylation pathway, through the action of various enzymes and vitamins, particularly folic acid (vitamin B9) and B12 used when intracellular methionine levels are low, thus restoring the necessary levels to correctly maintain active protein synthesis. A second pathway, used in cases of intracellular methionine excess, (the trans-sulfuration pathway) is the one that recycles Hcy into cysteine (a precursor of glutathione), first passing through cystathionine (via the enzyme cystathionine beta-synthase), a reaction that requires vitamin B6 in its active form. HHcy has been identified as a risk factor for a variety of disorders, including cardiovascular diseases, multiple sclerosis, diabetes, Alzheimer’s and Parkinson’s diseases, osteoporosis and cancer. However, it remains unclear whether the slightly elevated concentration of Hcy (Hcy 7–10 μmol/L) is a causative factor or simply a marker of these pathologies. In human plasma, the concentration of Hcy ([Hcy]) is classified as mild (15 to 30 μmol/L), moderate (30 to 100 μmol/L), and severe (greater than 100 μmol/L). Interestingly, many laboratories continue to consider 25 μmol/L as normal. This review seeks to examine the controversial literature regarding the normal range of HHcy and emphasizes that even a [Hcy] level of 10 μmol/L may contribute to the development of several diseases, aiming to discuss whether it would be appropriate to lower the threshold of HHcy normal values.

## 1. Introduction

**Figure 1:**
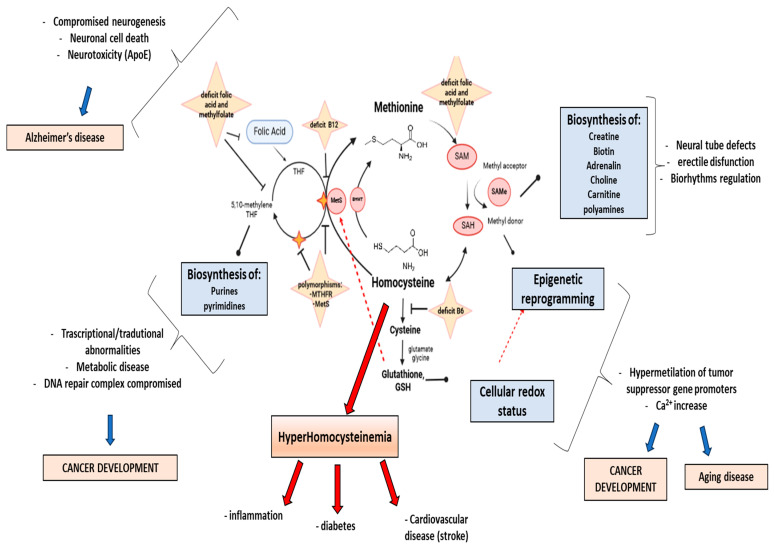
Schematic diagram of Hcy physiological production through the Meth and folate cycle. Dietary Meth is converted to Hcy through SAM and SAH and then back to Meth via the re-methylation pathway. Half of Hcy goes to the transsulfuration pathway, where it is converted to cysteine with the help of CBS. Then, cysteine is further converted to GSH; dietary folic acid (vitamin B9) enters the folate cycle after its conversion first to dihydrofolate (DHF) and then to THF. MTHFR is a key enzyme that converts 5,10-methylene-THF to 5-methyl-THF.

Epigenetics is the study of molecular modifications to DNA and chromatin that are heritable through cell division (mitosis) and potentially reversible, occurring without altering the underlying DNA sequence. Rather than altering the genetic code itself, these modifications dictate the timing, extent, and manner in which specific genes are expressed, functioning like a conductor that coordinates the harmony of gene expression. DNA methylation is indeed one of the most extensively studied epigenetic mechanisms. It involves the addition of a methyl group (-CH3) to the DNA molecule, typically at the 5′ position of cytosine rings, leading to 5-methylcytosine. This modification is crucial for various biological processes, including gene regulation, development, and genomic stability. It plays a role in a variety of diseases, including birth defects [1] and cancers [2,3], and can be influenced by environmental factors [4], behavior, and nutrition [5]. This epigenetic modification typically occurs at cytosine residues adjacent to guanine residues (5′-CG-3′), referred to as CpG sites, and plays a pivotal role in in both physiological and pathological conditions. Methionine (Meth) is an essential amino acid consumed from the diet which can be regenerated from homocysteine (Hcy) via methionine-synthase (MTR) in a reaction that requires vitamin B12 (cobalamin) as a cofactor. Disruption of MTR affects numerous methylation-dependent reactions, including epigenetic modifications, and also impacts the intracellular folate pathway. Figure 1 summarizes the key steps of the interlinked folate and Meth cycles, focusing on the main pathological and physiological downstream pathways (Figure 1).

One-carbon metabolism (OCM) refers to the biochemical processes that produce S-adenosylmethionine (SAM), which serves as the primary methyl donor for various activities, including DNA methylation [6]. During OCM, meth is metabolized to SAM by the methionine adenosyltransferase (MAT) family of enzymes (I, II, and III), supplying all of the methyl groups required for DNA methylation and protein post-translational modifications. In this regard, vitamin B6, folate (B9), and B12 are essential co-factors involved in many steps of OCM metabolism and in the degradation of Hcy either by remethylation or by transsulfuration [7,8]. Genetic factors, nutritional vitamin deficiencies, age and lifestyle factors may contribute to an abnormal increase in Hcy, leading to hyperhomocysteinemia (HHcy).

In such cases, the toxic effects of Hcy are at least partially due to oxidative damage to proteins and DNA [9], so efficient detoxification of Hcy is essential for maintaining genomic stability and cellular viability.

Thus, HHcy has been indicated as a risk factor for a variety of disorders, such as cardiovascular diseases [10,11,12], multiple sclerosis [11], diabetes [12], Alzheimer’s disease [13], osteoporosis and osteoporotic fractures [14] and cancer [15,16,17]. To date, it is unclear whether the slightly elevated concentration of Hcy is the causative agent or merely a marker for the pathology (Figure 1). However, Hcy is now recognized as a risk factor for a wide range of diseases and conditions, affecting individuals from conception to death. In human plasma, Hcy concentration ([Hcy]) is typically below 12–15 μM and the cysteine concentration level is 240–360 μM [18]. Yet, despite this, the widely acknowledged reference classification of HHcy is mild (15 to 30 μmol/L), moderate (30 to 100 μmol/L), and severe (greater than 100 μmol/L) [19]. Interestingly, numerous laboratories still persist in maintaining 25 μmol/L as normal.

This review aims to discuss the controversial literature about HHcy normality range and highlight that 10 μmol/L of [Hcy] may already prompt the development of several diseases.

## 2. The OCM Pathways

As already mentioned, Hcy is produced from Meth through a process called demethylation, which involves the formation of two important intermediates: S-adenosylmethionine (SAM) and S-adenosylhomocysteine (SAH). Under normal circumstances, Hcy is eliminated through three main pathways: (1) methylation to Meth, which can take place through the action of MTR; (2) through an irreversible transsulfuration pathway, in which Hcy is transformed into cystathionine and eventually into cysteine; (3) remethylation using glycine betaine (N,N,N-trimethylglycine, TMG) to Meth in the liver and kidneys via the enzyme betaine-homocysteine methyltransferase (BHMT), a Zn^2+^-dependent thiolmethyltransferase, and export to the plasma. Notably, BHMT comprises up to 1.5% of all the soluble protein of the liver, and recent evidence suggests that it may have a greater influence on Meth and Hcy homeostasis than MTR [20].

In the remethylation cycle, the metabolism of homocysteine is closely linked to the folate cycle, during which circulating folate is converted into tetrahydrofolate (THF). Upon undergoing methylation, THF is transformed into 5,10-methylene THF, which is subsequently reduced to 5-methyl THF by the enzyme methylene tetrahydrofolate reductase (MTHFR). This cycle is referred to as the remethylation cycle because it involves the conversion of Hcy back to Meth by incorporating a methyl group from 5-methyl THF derived from the folate cycle [21]. Thus, both folate and vitamin B12 are essential for Hcy remethylation.

Therefore, when Meth is sufficiently available, Hcy combines with serine and is then broken down into α-ketobutyrate and cysteine. Cysteine serves as a precursor for glutathione (GSH) and a deficiency in the transsulfuration pathway decreases GSH synthesis, the body’s primary antioxidant [8]. In addition to its antioxidant role, GSH also has anti-inflammatory properties, as it reduces interleukin production and the expression of TNF-alpha and iNOS synthase. However, insufficiency in the transsulfuration pathway also leads to HHcy. This condition diminishes the activity of cellular glutathione peroxidase (GPx1), an intracellular antioxidant enzyme that converts hydrogen peroxide to water, favoring GSSH over GSH. The resulting imbalance in the GSH/GSSH ratio contributes to various cardiovascular and neurodegenerative disorders. In both cases, N-Acetyl-Cysteine (NAC) supplementation provides the cysteine needed for GSH synthesis, reduces HHcy, enhances GPx1 activity, and further alleviates oxidative stress [22]. The transsulfuration pathway, which mainly involves cystathionine β-synthase (CBS), a key enzyme that converts Hcy to cystathionine, represents the metabolic link between antioxidant and methylation metabolism.

The primary cause of HHcy is genetic defects in the enzymes responsible for Hcy metabolism, a topic of significant scientific interest [23]. Specifically, polymorphisms in key enzymes involved in Hcy metabolism, such as MTHFR (the most common is the C677T polymorphism) [24], CBS (the most important polymorphism is T833C) [25], MTR [26] and methioninesynthase reductase (MTRR), have been identified as important subjects of study [27].

### 2.1. Homocysteine and Cardiovascular Disease

HHcy is emerging as a prevalent and strong risk factor for atherosclerotic vascular disease in the coronary, cerebral, and peripheral vessels, and for arterial and venous thromboembolism [28]. In fact, persistently high levels of Hcy contribute to the development of atherosclerotic plaques and increase the risk of atherothrombotic events by inducing endothelial dysfunction and exacerbating inflammation, as well as creating a thrombophilic profile. Due to these effects, both the World Health Organization (WHO) and health ministries have acknowledged HHcys as a significant risk factor for cardiovascular disease (CVD), alongside traditional risk factors [29]. The mechanisms by which Hcy may contribute to the development of CVD are now well established, such as its adverse effects on the vascular endothelium and smooth muscle cells, which lead to alterations in subclinical arterial structure and function. Therefore, HHcy is considered an independent risk factor for atherosclerosis [30,31] and several studies clearly define a strong correlation between elevated Hcy and myocardial infarction, stroke and increased cardiovascular mortality [32]. Hcy affects blood vessels by regulating the contractility of vascular smooth muscle cells and the permeability of endothelial cells. The major mechanism involved is the inhibition of endothelial nitric oxide synthase (eNOS), which is responsible for producing nitric oxide (NO) [33,34]. Under normal conditions, a system of antioxidant mechanisms regulates the production of reactive oxygen species (ROS). However, during adverse and chronic conditions, an imbalance in this system disrupts the generation of both NO and ROS, resulting in endothelial dysfunction [35]. Oxidative stress, thiolactone formation and protein homocysteinylation are directly related to endothelial toxicity [36,37]. In vitro studies have proposed two main mechanisms by which Hcy contributes to the accumulation of reactive oxygen species (ROS): by decreasing the activity of GPx1 and by inhibiting dimethylarginine dimethylaminohydrolase (DDAH), an enzyme involved in the metabolism of asymmetric dimethylarginine (ADMA), which is an endogenous inhibitor of eNOS [33,37]. Additionally, HHcy-induced oxidative stress is known to activate matrix metalloproteinases (MMPs), which disrupt extracellular matrix (ECM) metabolism and increase collagen deposition, leading to vascular fibrosis [38]. Therefore, the proinflammatory effect of HHcy is linked to ROS generation and involves the activation of nuclear transcription factor κB (NF-κB), which regulates mainly the genes responsible for the expression of intercellular adhesion molecule-1 (ICAM-1), monocyte chemoattractant protein-1 (MCP-1), vascular adhesion molecule-1 (VCAM-1) and E-selectin, leading to the progression of atherosclerosis [39,40,41]. The role of Hcy in the activation of factor V and tissue factor (TF), which propagate coagulation and the parallel inhibition of antithrombin III, has also been well established [42,43].

Several studies in adults indicated that the risk of coronary artery disease is directly linked to [Hcy], with significant risk observed between 10 and 15 µmol/L. Furthermore, for every 5 µmol/L increase in [Hcy], the risk increases by nearly 20% [44,45]. In the Third National Health and Nutrition Examination Survey (NHANES) conducted from 1988 to 1994, researchers found that serum [Hcy] levels were independently associated with blood pressure. Specifically, a 5 μmol/L increase in Hcy was associated with an increase in diastolic blood pressure of 0.5 and 0.7 mm Hg and an increase in systolic blood pressure of 0.7 and 1.2 mm Hg in men and women, respectively. These findings were based on a sample size of 5978 participants [46]. Current guidelines do not recognize Hcy as a CVD risk stratification tool, even though prospective studies showed that rising Hcy levels predict adverse CV events better than the Framingham Risk Score, suggesting it could be considered a “novel” CVD risk marker [47]. Carnagarin and colleagues demonstrated that in hypertensive patients with Hcy above 10 µmol/L, ACE inhibitors may be less effective in reducing blood pressure and preventing vascular damage [48] (Table 1).

### 2.2. Homocysteine and Neurodegenerative Diseases

#### 2.2.1. Multiple Sclerosis (MS)

The manipulation of methylation processes has been shown to affect the vulnerability of neurons to degeneration and apoptosis in experimental models of neurodegenerative disorders such as Alzheimer’s disease (AD) [49], Parkinson’s disease (PD) [50] and Huntington’s disease (HD) [51]. Specifically, changes in the DNA methylation patterns, which are important regulators of gene expression, have been implicated in the pathogenesis of neurodegenerative diseases [52]. Modulating these epigenetic modifications can influence the susceptibility of neurons to the detrimental effects that lead to neurodegeneration and cell death. Indeed, SAM is also involved in the methylation of proteins, phospholipids and neurotransmitters [53], suggesting a role for this enzyme in signal transduction processes in the nervous system. The accumulation of Hcy can directly damage and kill neurons and some in vitro studies showed that the toxicity is caused by the activation of N-methyl-D-aspartate (NMDA) receptor [54] or apoptosis triggered by DNA damage [49], which typically involved PARP and p53 activity.

Multiple sclerosis (MS) is a chronic inflammatory demyelinating disease of the central nervous system (CNS) that results in neurological disability. The etiology of MS is thought to be multifactorial, with genetic and environmental factors interacting to contribute to the autoimmune inflammatory process [55]. Numerous studies have examined the potential roles of Hcy, vitamin B12, and folate as contributors to the neurodegenerative process [55].

Indeed, HHcy may be a risk factor for neurological decline in MS and may have a neurodegenerative effect [56]. Several studies have reported a significant increase in plasma [Hcy] in MS patients compared to healthy controls. However, the precise mechanism by which elevated Hcy contributes to the pathogenesis of MS is not yet fully understood [57]. However, the key condition in the development of MS is the dysregulation of the blood–brain barrier (BBB) and the increased migration of leukocytes across the BBB [58]. In particular, the SOD1G93A transgenic mouse model of amyotrophic lateral sclerosis (ALS) demonstrated that the level of 5-5-methyltetrahydrofolate (MTHF) significantly decreased in the plasma, spinal cord and cortex at the early stages of pre-symptomatic ALS and that the levels of Hcy were markedly elevated even after the motor symptoms appeared [59].

In humans, the levels of Hcy found in the cerebrospinal fluid (CSF) and brain tissue are reported to range from 0.5 to 10 μm [60] and can be taken up rapidly by neurons via a specific membrane transporter (L-homocysteine sulphinate (L-HCSA) and L-homocysteate (L-HCA)) [61]. It has been demonstrated that Hcy levels increased up to 20–30 μm in amyloid precursor protein (APP) mutant transgenic mice maintained on the methyl donor-deficient diet, while folic acid supplementation seemed to reduce the risk of sporadic forms of AD and also might suppress the neurodegenerative process [62]. Interestingly, even after adjusting for vitamin B6, vitamin B12, and folate, patients with MS had a statistically significant higher plasma [Hcy] (4.5 μmol/L, 6.2 vs. 2.7 μmol/L) compared to healthy patients, indicating that the elevated levels might be due to increased production rather than decreased removal [63] (Table 1).

#### 2.2.2. Alzheimer’s Disease (AD)

Alzheimer’s disease is a progressive and neurodegenerative disorder with typical symptoms of progressive memory loss and cognitive dysfunction [64] closely associated with a variety of risk factors such as aging, ApoE4 genotype, hyperglycemia and HHcy [65]. A much more consistent number of studies indicated that HHcy is an important and independent risk factor and biomarker independent of B vitamins for AD [66,67]. Recently, a meta-analysis (28,257 participants) found that every 5 μmol/L increase in blood Hcy is linearly associated with a 15% increase in relative risk of Alzheimer-type dementia [68]. It has been reported that in patients with AD, Hcy levels do not increase over time following the onset of the disease; however, these levels correlate with the severity of disease [49]. Additionally, it is suggested that dementia may develop prior to HHcy in the later stages [69].

In in vitro studies conducted on cultured neurons, Hcy activated tau phosphokinases (glycogen synthase kinase 3 and cyclin-dependent kinase 5) and inhibited protein phosphatase 2A (a main tau phosphatase), leading to increases in phosphorylated tubulin associated unit (TAU) levels, as well as increases in tau aggregates and truncated TAU species [70]. Accordingly, in vivo studies demonstrated that high doses of Hcy (16.4 mmol/L vs. 8 mmol/L in control group), induced by diet in widely used mice containing three mutations (3XTg mice) associated with familial AD, were able to promote TAU phosphorylation at T231/S235 sites.

Some cohort studies have suggested that higher amounts of Hcy, Meth, and SAM may accelerate cognitive decline in patients with mild cognitive impairment (MCI) or AD, and that vitamin B12 deficiency may worsen the clinical outcome [71]. An interesting case series demonstrated that decreased levels of vitamin B12 and folate and serum [Hcy] from 22.5 to 8 µmol/L directly correlate with atrophic changes in the cerebral cortex and AD progression [72] (Table 1).

Moreover, the normalization of HHcy has been shown to enhance cognitive function and reduce brain amyloidosis in a transgenic mouse model of AD [73].

### 2.3. Homocysteine and Diabetes Mellitus (DM)

HHcy has been implicated in the pathogenesis of DM, owing to its promotion of oxidative stress, β-cell dysfunction, and insulin resistance [74,75]. Recent in vivo and in vitro studies indicate that OCM nutrients play a crucial role in supporting energy and glucose metabolism through various mechanisms. Indeed, low levels of folate, choline, or vitamin B12 have been shown to induce HHcy, which is linked to the development of DM [76,77]. As mentioned above, HHcy elevates ROS and C-reactive protein (CRP) levels, promoting oxidative stress and systemic inflammation. The activation of stress-sensitive signaling pathways can ultimately result in pancreatic β-cell dysfunction [78], glucose intolerance, and insulin resistance [79]. Changes in Hcy metabolic enzymes have been observed in different diabetic conditions [80,81]. In rats on a high-fat sucrose diet, there was a positive correlation between plasma insulin levels and both Hcy and MTHFR activity, while an inverse correlation was found with cystathionine-β-synthase (CβS) activity [82,83]. Indeed, some in vitro studies showed that folic acid supplementation during the rat juvenile–pubertal period increased methylation in the promoter regions of insulin receptor, peroxisome proliferator-activated receptor-α (PPAR-α) and glucocorticoid receptor genes involved in metabolic homeostasis [84]. Analogously, mice that were deprived of folate in utero exhibited hypomethylation at the differentially methylated region (DMR) 1 of the imprinted locus insulin-like growth factor 2 (Igf2), as well as at Slc389a4CGl1, in blood, liver, and kidney tissues [85].

HHcy is directly linked to a greater risk of developing type 2 diabetes mellitus (T2DM) [77]. Thus, a cross-sectional study performed on a cohort of 75 patients with T2DM and 54 healthy control subjects clearly demonstrated that plasma Hcy was significantly higher in patients with T2DM compared with controls (Hcy 12.0 ± 0.7 vs. 8.7 ± 0.3 μmol/L) [86] and that insulin resistance and renal function are independent determinants of Hcy levels in DM patients. Recent evidence showed that in subjects >65 years with DN (n = 1845) and in a non-DM group (n = 28,720), concentration of Hcy > 12 µmol/L was a good indicator to predict impaired kidney function in DN patients and that [Hcy] in DN was inversely proportional to the estimated glomerular filtration rate (eGFR) [87]. Therefore, some clinical trials demonstrated that elevated Hcy levels (>50 mol/L) and/or fenofibrate therapy, one of the best options to treat the atherogenic lipid triad (i.e., high triglycerides, low HDL, elevated small-size LDL) of T2DM, may interfere with other atheroprotective functions of HDL particles such as anti-oxidative and anti-inflammatory actions. In particular, HHcy markedly impaired the ability to facilitate cholesterol efflux from macrophage foam cells independently of apoA-I levels [88]. Moreover, high plasma [Hcy] has been associated with increased risk for coronary heart disease (CHD) events in DM individuals. Notably, T2DM patients with plasma Hcy of 15 µmol/L or more at baseline had a higher risk for CHD death than those with plasma Hcy levels less than 15 µmol/L (26.1% and 13.5%, respectively; *p* = 0.005) [89] (Table 1). Another significant issue that affects individuals with both type 1 and type 2 diabetes is retinopathy, a specialized neurovascular complication. Brazionis and colleagues observed that HHcy is strictly correlated with retinopathy in TD2M individuals with a mean plasma [Hcy] of 11.5 mumol/L vs. 9.6 mumol/L of diabetic patients without retinopathy. The authors concluded that a slight rise in plasma Hcy levels, around 1 μmol/L, could serve as a valuable signal to enhance the treatment of key risk factors for diabetes complications such as blood pressure, blood glucose, and blood lipids [90]. The likelihood of developing this condition is closely linked to how long a person has had diabetes and how well their blood sugar levels are managed.

### 2.4. Homocysteine and Osteoporosis

Osteoporosis is a major health problem characterized by low bone mineral density (BMD), deterioration of bone microarchitecture, and increased risk of fracture [91]. Osteoporotic fractures (OF) are associated with increased morbidity and mortality and substantial economic costs [92,93,94]. Recent investigations have revealed a link between elevated plasma Hcy levels and both lower BMD and a higher risk of bone fractures [14,95,96]. HHcy is now recognized as an independent risk factor for osteoporosis and fractures, particularly in the elderly [97].

Two independent prospective studies examined the relationship between Hcy levels and fracture incidence in three groups of men and women aged 55 years and older. The observed association between Hcy levels and fracture risk was primarily related to BMD, as well as dietary factors such as calorie, protein, calcium, and vitamin intake [14]. This study involved 2406 individuals and found a significant association between circulating Hcy levels and the risk of OF (RR = 1.4 per 1 SD increase in natural log-transformed Hcy level; 95% CI, 1.2–1.6) [14]. Furthermore, elevated Hcy levels (>20 μmol/L for men and >18 μmol/L for women) were associated with a substantial increase in fracture risk (4.1-fold for men and 1.9-fold for women) [98] (Table 1).

Several mechanisms have been proposed to explain the connection between HHcy and the development of osteoporosis: (i) decreased bone formation due to apoptosis in human bone marrow stromal cells [99], (ii) stimulation of osteoclastogenesis through increasing intracellular radical oxygen species (ROS) generation and activation of matrix metalloproteinases (MMPs) that can then degrade extracellular bone matrix [100], (iii) reduced bone blood flow, leading to changes in bone biomechanical properties [101], (iv) altered gene expression with reduced methylation capacity, (v) and reduced bone strength through interference with collagen cross-links [96].

Hcy was shown to induce apoptosis in primary human bone marrow stromal cells and the HS-5 cell line, and this apoptotic effect was caspase-dependent. Furthermore, Hcy induced an increase in the release of cytochrome c into the cytosol and activated caspase-9 and caspase-3, but not caspase-8, indicating that the induction of apoptosis occurred through the mitochondrial pathway [99].

HHcy significantly reduced bone blood flow, as demonstrated in studies where Hcy-treated rats exhibited a lower tibial blood flow index compared to controls [102], and this reduction in blood flow was associated with increased oxidative stress and MMP activity, leading to extracellular matrix degradation [100]. HHcy is also linked to altered gene expression through reduced DNA methylation capacity, impacting various cellular processes, mainly by increasing the expression of 5-lipoxygenase (5LO) and subsequently inducing hypomethylation of its promoter, which is associated with elevated S-adenosylhomocysteine (SAH) levels and reduced DNA methyltransferase activity [103]. Chronic HHcy exposure can also lead to the demethylation of the human telomerase reverse transcriptase (hTERT) promoter, resulting in decreased telomerase activity and accelerated senescence in endothelial cells. This process is mediated by the repression of key transcription factors and reduced DNA methyltransferase expression [104,105]. While the predominant view highlights Hcy’s detrimental effects on gene expression via hypomethylation, some studies suggest potential compensatory mechanisms, such as dietary interventions that may restore methylation capacity and mitigate these effects [106].

Blouin and colleagues investigated the relationship between plasma Hcy levels and bone material properties, specifically focusing on collagen cross-link ratios in patients who had sustained femoral neck fractures [96]. In trabecular areas undergoing bone formation, the collagen cross-link ratio was significantly higher in the high-Hcy group (7.6 ± 0.2; N = 10) compared to the low-Hcy group (6.4 ± 0.2; N = 9). Conversely, in areas undergoing resorption, there was no significant difference in the collagen cross-link ratio between the two groups (12.5 ± 0.5 for high Hcy and 11.4 ± 0.4 for low Hcy). A strong correlation was found between blood Hcy levels and collagen cross-link ratio at forming trabecular surfaces (Spearman’s r = 0.83, *p* < 0.0001), but not at resorbing surfaces (Spearman’s r = 0.45, *p* > 0.05). The study concluded that while there is a significant correlation between plasma Hcy levels and collagen cross-link ratios in bone-forming areas, this relationship is independent of bone mineral content and distribution patterns [96].

Although the relationship between Hcy levels and osteoporosis is increasingly recognized, future research should focus on larger cohorts and explore potential therapeutic interventions, such as probiotics, to mitigate the detrimental effects of Hcy on bone health. While current findings suggest a significant association between Hcy and osteoporosis, the lack of consensus on causality calls for further studies to clarify these relationships and explore potential treatment avenues. Therefore, in clinical practice, it is essential to identify patients with potential causes for elevated homocysteine levels (such as certain medications). These patients may benefit from vitamin D and folic acid supplementation as needed. This strategy could enhance not only bone health but also vascular and overall well-being [107].

### 2.5. Homocysteine and Cancer

Cancer cells exhibit a strong reliance on the Meth cycle, leading to the production of significant amounts of Hcy. Elevated intracellular Hcy levels can be released into the bloodstream, prompting numerous studies to establish a link between high Hcy levels and cancer [16]. Numerous laboratories classified blood Hcy levels exceeding 15 μmol/L as HHcy, which is associated with oxidative stress, endoplasmic reticulum (ER) stress, apoptosis, protein oxidation, inflammation, and impaired angiogenesis [108,109]. Moreover, several studies have reported inconsistent associations between polymorphisms in genes related to Hcy metabolism and cancer [15,110,111], but together with dietary Meth, folate, vitamin B12, B6, and alcohol consumption, genetic polymorphisms maybe responsible of tumor genesis. These polymorphisms are often linked to HHcy and different cancer types. The most common mutations in MTHFR 677C->T transition at codon 222 and 1298A->C transversion at codon 429 have been associated with cervical [112], colorectal [113], endometrial [114], and esophageal cancer [115]. Analogously, MTRR gene A66G Ile22Met is found to be associated with colorectal cancer (CRC) [116] and leukemia [15]. Likewise, the allele of MTR2756G showed a positive association with CRC and the association with MTHFR1298AA further increases the risk of developing this cancer type [117].

Therefore, strong evidence supported an association between plasma Hcy levels and progression of rectal cancer from normal, to rectal adenoma, to rectal cancer. Therefore, homocysteine (Hcy) could potentially serve as a therapeutic tool to assist clinicians in the staging of rectal cancer. In fact, plasma [Hcy] directly increased from normal control subjects to patients with low-risk and high-risk adenomas, and to patients with Stage I–IV rectal cancer [118]. Similarly, from a recent metanalysis, it was found that every 5 μmol/L increase in Hcy was associated with a 7% higher risk of digestive cancer occurrence and the authors concluded that [Hcy] may be a potential biomarker in this type of cancer [119] (Table 1).

**Table 1:** Range values of Hcy in diseases. The table summarizes the range of Hcy values associated with each disease covered in the review.

Disease	Plasma [Hcy]	REF
Cardiovascular disease	every 5 µmol/L increase in [Hcy], the risk of CD increases by nearly 20% <10 µmol/L (coronary artery disease) >10 µmol/L (ACE inhibitors failure)	[45,46] [45,46] [49]
Neurodegenerative diseases (MS and AD)	every 5 μmol/L increase in blood homocysteine is linearly associated with a 15% increase in relative risk of AD 4.5–6.2 μmol/L MS patients vs. 2.7 in healthy patients From 8 to 22 µmol/L directly correlates with atrophic changes in the cerebral cortex and AD progression	[69] [64] [73]
Endocrine disease: (DM) Osteoporosis	<15 µmol/(CHD death in DM patients) DM patients 12.0 ± 0.7 vs. healthy patients 8.7 ± 0.3 μmol/L Hcy > 12 µmol/L was a good indicator to predict impaired kidney function in DM patients 1 µmol/L increase in Hcy was correlated with a 15% to 20% increase in the risk of diabetic retinopathy >20 μmol/L(men) and >18 μmol/L(women) increase fracture risk	[86] [83] [84] [90] [96]
Cancers	every 5 μmol/L increase in homocysteine was associated with a 7% higher risk of digestive cancer	[116]

Increasing evidence suggests that variations in the use of dietary nutrients significantly influence tumor metabolism, growth, and treatment results [120,121,122]. One-carbon metabolism, which plays a critical role in redox and nucleotide metabolism, is a key target for standard cancer treatments like 5-fluorouracil and radiation therapy [123,124]. Particularly, Meth is one of the nutrients that cancer cells require to maintain cell proliferation, survival, and metastasis [125]. Cells employ various pathways to convert folic acid and 5-methyl THF into THF, which can then accept a one-carbon unit for subsequent biosynthetic reactions. Folic acid is converted to THF by the enzyme dihydrofolate reductase (DHFR), a known target of the antifolate chemotherapy drug methotrexate. On the other hand, the conversion of 5-methyl THF to THF is linked to the synthesis of Meth. This process involves the transfer of a methyl group to Hcy, facilitated by the vitamin B12-dependent enzyme methioninesynthase (MTR). MTR is the sole enzyme recognized for using 5-methyl THF as a substrate to regenerate the THF structure. Consequently, a deficiency in cobalamin leads to a loss of MTR activity, resulting in an accumulation of intracellular 5-methyl THF, a phenomenon clinically referred to as the “methyl-folate trap”. This irreversible accumulation of folates as 5-methyl THF limits the availability of THF in cells for new one-carbon unit incorporation. Several lines of evidence suggest that MTR’s role in the folate cycle may be critical for tumor growth [126].

A study revealed that cancer cells exhibit elevated levels of the Meth transporter SLC43A2, facilitating their increased uptake of Meth, which in turn promotes cancer progression [127]. Growing evidence suggests that dietary interventions can effectively be used as therapeutic tools for treating cancer. In this regard, lowering the intake of Meth or cystine has been shown to safeguard non-tumor cells and enhance the immune surveillance system in the fight against cancer [128]. Indeed, in an in vivo study, dietary Meth restriction (MR) has been shown to increase [129] and enhance metabolic health [130]. Notably, certain cancer cell lines are dependent on Meth for growth [131] and limiting or reducing Meth intake in the diet may exhibit anti-cancer properties in mouse models of colon carcinogenesis [132].

## 3. Discussion

The range of diseases associated with HHcy is fairly well delineated. Indeed, the relationships between Hcy and CV, neurological, endocrine and oncological diseases revolve around its biochemical pathways. As mentioned above, Hcy is a toxic sulfur-containing amino acid that results from Meth metabolism, and its levels are influenced by various factors, including genetic, nutritional, and lifestyle elements. HHcy has been listed by the World Health Organization (WHO) as a cardiovascular, cerebrovascular and peripheral vascular risk factor [133], but its correlation with dysmetabolic disease and cancer is likewise disputed. In particular, there is a lot of confusion about the definition of optimal reference ranges. Actually, regarding reference ranges, it is recommended that each laboratory center conducting the analysis develop its own reference intervals for [Hcy]. This task is complicated by numerous variables that influence circulating concentrations, including age, gender, ethnicity, pregnancy, menopause, smoking status, nutritional status, renal function, medication use, and the fortification of food with folic acid. For adults, the reference range for Hcy is generally between 6 and 10 μmol/L; however, in countries where food fortification with folic acid is mandated, this range is typically 10–30% lower. In 2003, a consensus document defined HHcy based on plasma concentrations, categorizing it as moderate (Hcy levels of 15–30 μmol/L), intermediate (30–100 μmol/L), and severe (greater than 100 μmol/L). So, Hcy is defined as normal when <15 μmol/L [134]. Nowadays, many laboratories continue to utilize these reference ranges, despite the fact that they are outdated and do not align with contemporary scientific literature. As discussed in this review, in CVD and AD patients, the normality threshold is much lower. Indeed, many studies indicated that Hcy < 10 μmol/L is recommended and that a significant increase in CVD and neurological disorder risk is observed precisely between 10 and 15 μmol/L [10,30,31,48,135]. The scientific community concurs that even a 5 μmol/L rise in Hcy levels serves as a predictor for various diseases, including cardiovascular, endocrine, neurodegenerative and oncological conditions. Indeed, a dose–response meta-analysis conducted in 2017 demonstrated a linear relationship between Hcy levels and the risk of all-cause mortality, indicating that for each 5 µmol/L increase in Hcy, there is a corresponding 33.6% rise in the risk of all-cause mortality [8,44,68,118].

## 4. Methods

For this narrative review, we searched English-language publications in PubMed published mostly in the last ten years (2014–2024). Our main search terms, their associations and their acronyms were “homocysteine, cardiovascular disease, diabetes mellitus, cancer, osteoporosis, neurodegenerative diseases”. In this narrative review, we considered the role of Hcy in the above-mentioned diseases focused on the plasma [Hcy]. English-language articles related to the topic were considered if they discussed one of the issues of interest and were peer reviewed.

## 5. Conclusions

Hcy metabolism is a complex network of pathways involving numerous enzymes, cofactors, and regulatory mechanisms. These pathways facilitate the conversion of Hcy to Meth or its degradation through transulfuration, maintaining a delicate balance crucial to health. Disruption of this system and the toxic accumulation of Hcy plays a key role in the onset and progression of various disease conditions. In this intricate scenario, a critical role is played by HHcy, which can affect the onset and progression of several pathological conditions such as cardiovascular diseases, endocrine pathologies, neurodegenerative pathologies, osteoporosis and cancer. Despite its importance, confusion persists in defining optimal reference intervals for Hcy, which limits accurate risk assessment and early diagnosis of Hcy-associated diseases. We believe that revised criteria should allow people to be included in risk groups for developing diseases and be more frequently and carefully monitored to avoid disease onset. The suggested threshold limit of 10 μmol/L could assist clinicians in identifying individuals at risk more promptly, allowing for appropriate and timely preventive interventions.
